# Clinical and Surgical Management of an Aggressive Cherubism Treated with Autogenous Bone Graft and Calcitonin

**DOI:** 10.5402/2011/340960

**Published:** 2010-10-17

**Authors:** Mônica Fernandes Gomes, Lilibeth Ferraz de Brito Penna Forte, Cybelle Mori Hiraoka, Flávio Augusto Claro, Mônica Costa Armond

**Affiliations:** ^1^Bioscience Center for Special Health Care Needs (Centro de Biociências Aplicado a Pacientes com Necessidades Especiais/CEBAPE-UNESP), São José dos Campos Dental School of the São Paulo State University (UNESP), São José dos Campos, São Paulo, Brazil; ^2^Special Health Care Needs Association (Associação Pró-Saúde para Pacientes com Necessidades Especiais-ASPE), São José dos Campos, São Paulo, Brazil; ^3^Centro de Biociências Aplicado a Pacientes com Necessidades Especiais-CEBAPE/UNESP, Av. Eng. Francisco José Longo, 777, São José dos Campos, São Paulo, Brazil; ^4^São José dos Campos Dental School of the São Paulo State University (UNESP), São José dos Campos, São Paulo, Brazil; ^5^Private Practice in Oral and Maxillofacial Surgery and Prosthesis, Taubaté, SP, Brazil; ^6^Vale do Rio Verde de Três Corações University (UNINCOR), Minas Gerais, Brazil

## Abstract

Cherubism is a rare autosomal-dominant inherited syndrome and is usually self-limiting; it starts in early childhood and involutes by puberty. It is a benign fibroosseous disease, characterized by excessive bone degradation of the upper and lower jaws followed by development of fibrous tissue masses. The purpose of this clinical report is to describe a rare and aggressive form of cherubism on an adult female patient that has been treated in our Bioscience Center for Special Health Care Needs-CEBAPE. The patient was firstly submitted to the surgical procedure with partial curettage of the lesion, and the cavity was filled with autogenous cancellous bone and bone marrow grafts. Furthermore, the support treatment used was the administration of salmon calcitonin by nasal spray during the first year after the preconized procedure. At 4-year followup, we confirmed the stomatognathic system improvement and esthetic rehabilitation, which led to a significant increase in the patient's quality of life.

## 1. Introduction

Cherubism is an inherited condition characterized by bone degradation and replacement by fibrous tissue at maxilla and mandible during childhood. This disease tends to show variable degree of remission or spontaneous involution after puberty; nevertheless, some facial deformity may persist. In rare cases, the disease remains active during adulthood [1–8]. The incidence is unknown, and the age of onset is between 2 and 10 years old [5, 6, 8]. This abnormality is a familial disease in which the trait is transmitted as an autosomal dominant pattern, although several sporadic cases with no detectable family history have been already described [9], which may be due to incomplete penetrance or new mutations [3].

Cherubism is caused by seven mutations in the gene encoding SH3-binding protein SH3BP2 on chromosome 4p16.3 [2, 4]. The SH3BP2 mutation is thought to lead to parathyroid hormone receptor (PTHr) signaling and Msx-1 activation. The gain-of-function mutation results in a compartmentalization failure of the cap stage during molar development leading to deregulation of bone formation and remodelation, development of multinucleated giant cells, and abundant deposition of fibrohistiocytic tissues [4]. The penetrance is approximately 80% (100% in males and 50% to 70% in females), although the accurate estimate will depend on whether or not, clinical or radiological diagnostic criteria have been used [6, 9–11].

The differential diagnosis for cherubism includes four conditions: hyperparathyroidism, central giant-cell tumor, ossifying fibroma, and fibrous dysplasia of the jaw [9, 10, 12–14].

The physical examination of the patient affected with cherubism demonstrate a large spectrum of clinical appearances, therefore, the correct diagnosis and treatment plan depend on a well-collected data regarding the location of the lesion, as well its histopathological and radiological features. Even though, the disease has been researched, the medical and dentistry community has not established a consensus to the best form of treatment for cherubism [6, 8]. Some researchers have studied the effect of calcitonin treatment in bone metabolism and its therapeutic values for treatment of central giant cell granuloma [15, 16]. This hormone inhibits bone resorption, acting directly in the osteoclastic cells [3, 4, 17], whereas other works highlight the importance of an autogenous bone graft as an osteoinductive implant material [18–21]. As seen that, the chemotactic, mitogenic, and osteogenic potential of the autogenous bone and bone marrow grafts associated to the presence of growth factors and mesenquimal stem cells, have been reported by Lieberman et al. [19] and Szpalski and Gunzburg [20] have been reported that autogenous bone and bone marrow grafts have a chemotactic, mitogenic, and osteogenic potential. 

In this present clinical study, we reported a case of one highly debilitating aggressive cherubim in a young adult patient, submitted to surgical procedure. The laboratorial test results and the clinical, radiographic, and histological features were analyzed, and we correlated these findings with those of the literature. Additionally, the patient was followed-up for 4 years in our Research Center after the adopted treatment protocol, which consisted of partial curettage of the lesion, immediate implantation of autogenous cancellous bone plus bone marrow grafts into the surgical bone defect, and administration of systemic salmon calcitonin during the first year postoperative.

## 2. Case Report

The patient under study is an 18-year-old female leucoderm patient who presented highly invasive, expansive, and osteolytic lesion. Clinically, bilateral enlargement of the face, mouth opening limited to approximately 2.0 cm, and bilateral eversion of lower vestibule were detected. The total eruption of the right lower third molar, the partial eruption of the left lower third molar, and total eruption of the upper left and right third molars were evidenced. The oral mucosa of the patient at these regions showed normal aspects. 

**Figure 1:**
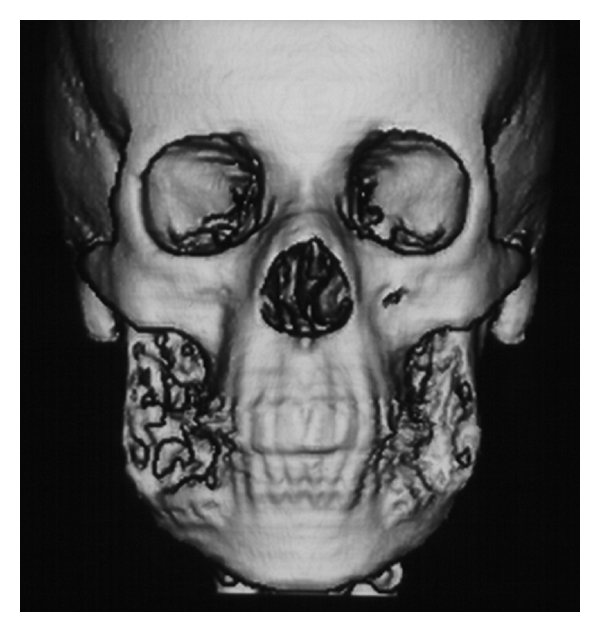
3D reconstruction of the CT scan images showing erosive aspect and multiple depressions at the bilateral vestibular cortical bone of the retromolar region (Gomes et al. [6]).

**Figure 2:**
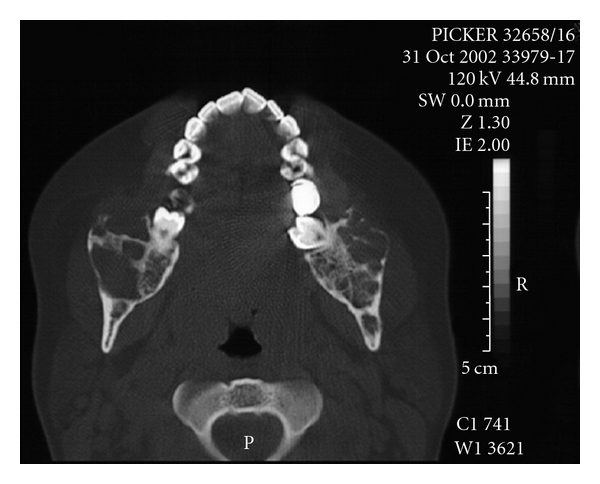
Axial CT scan revealing large hypoattenuated heterogenous image with aspect of expansive and invasive, osteolytic, multilocular lesion with perforation of the cortical bone at the angle and ramus of the mandible (Gomes et al. [6]).

**Figure 3:**
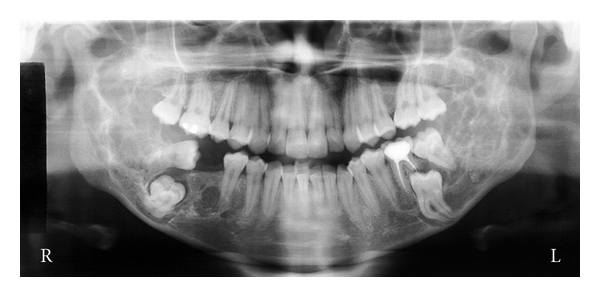
Panoramic radiograph showing bilateral growth of the lesion in the body, ramus, and coronoid process of mandible, atypical eruptions of the lower third molar, and resorption of the distal root of the left lower first molar (Gomes et al. [6]).

The 3D reconstruction at the CT scan views, showed a thin and/or eroded area in the vestibular and lingual cortical bones of the retromolar region, exhibiting multiple deep depressions, occurring mainly and more extensive on the right than on the left side. However, there was an absence of the lesion bone in the condyle processes, and the cortical bone of the mandibular basis showed normal features (Figure 1). The axial CT scan and the panoramic radiograph showed large and a soap bubble-like cavities at the angle and ramus of the mandible, as well as thin or disrupted vestibular mandibular cortical bone (Figures 2 and 3). This lesion spread from the premolar toward the coronoid process in the right and from the molar toward the coronoid process in the left side. Moreover, there were no signs of the lesion in the maxilla, and the lower second molars remained surrounded by the lesion.

The results of the laboratory tests were within normal limits for alkaline phosphatase (20 lU l-I; normal: adults 13–43 lU l-I, child 56–156 lU l-I), calcium (8.6 mg dI-I; normal: 8.8–11.0 mg dl-1), phosphorus (4.1 mg dI-I; normal: adult 2.5–4.8 mg dI-I, child 3–7 mg dI-I), and parathyroid hormone levels (21.30 pg ml-I; normal: 13–54 pg ml-I).

**Figure 4:**
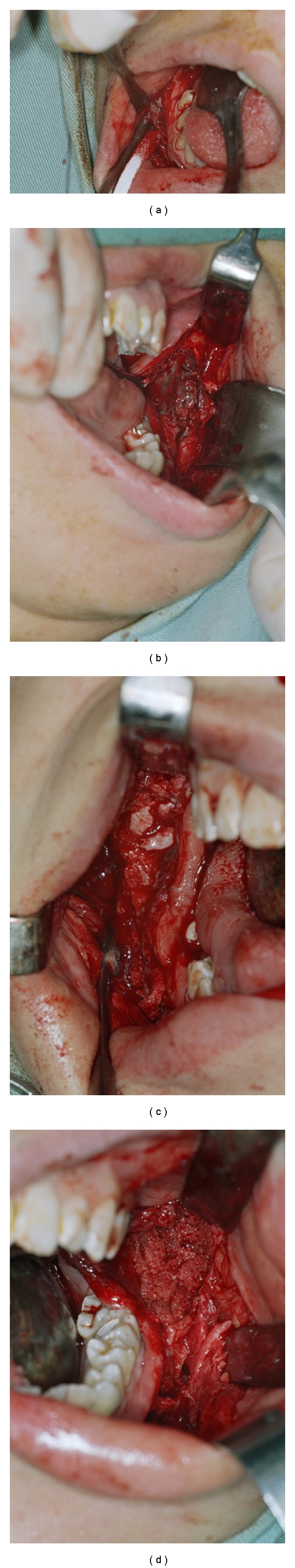
Right (a) and left (b) sides of the mandible showing lesion and the cavities without lesion filled by autogenous cancellous bone and bone marrow graft (c) and (d), respectively.

An incisional biopsy in the left mandibular area of the lesion was performed, and histological sections showed connective tissue looser and fewer multinucleated giant cells than the first histological examination done. Discrete number of blood vessels, various bone trabeculae, and osteoid tissue were also present (Figures 4(a) and 4(b)). The clinical, radiographic, laboratory, and microscopic features of the lesion suggested a diagnosis of diagnosis of Agressive Cherubism. 

The preconized treatment was partial curettage of the lesion, because the total removal could result pathological fractures and some bone cavities did not present surgical access. The multilocular cavities without lesion were totally filled by autogenous cancellous bone and bone marrow grafts (Figures 4(c) and 4(d)), obtained from the patient's iliac crest. The supported therapy in the postoperative was the administration of antibiotic Cefadroxil monohydrate 500 mg capsules (Cefamox, Bristol-Myers Squibb, São Paulo, Brazil), during ten days. Immediately after the surgical procedure, the patient was prescribed a systemic treatment using daily calcitonin in the form of salmon calcitonin nasal spray (Miacalcic, Norvatis AG, Switzerland), alternating the nostrils, in the dosage of 200 UI during the first year postoperative.

After surgical procedure, the patient was submitted to radiographic examinations annually until the remission of the lesion. 

After surgery, the laboratorial results remained normal to calcium (10.4 mg dl^−1^), phosphorus (4.1 mg dl^−1^), parathormone levels (22.9 pg mg^−1^), and calcitonin (2.0 pg mg^−1^; normal/women: <5.0 pg mg^−1^); however, there was an increase in the alkaline phosphatase (54 IU l^−1^), probably due to progressive bone formation process.

The patient presented an expressive Postoperatory improvement, which was preserved during all annual follow-up assessments. These periodicals controls will be done until the remission of the lesion. If the lesions do not go into a complete spontaneous remission, a complementary surgery could be performed. 

A future appointment will be scheduled for the extraction of the remaining unerupted teeth in order to avoid further development of cyst and/or odontogenic tumors.

## 3. Discussion

The cherubism's treatment depends on the rate of progression of the lesion, extension of tissues involved, and emotional state of the patient. The general recommendation from previous studies is to postpone the treatment until after puberty, unless severe psychological and physiologic dysfunctions impose an early intervention [2, 3, 10]. The literature review highlights a couple of considerations to be made when considering the treatment plan of an aggressive appearance of the cherubism. As reported by several authors, depending on the aggressiveness of the cherubism, can occur occur perforation of the cortical bone, weakening the area, which becomes more susceptible to fractures and/or infections [2, 10, 11]. Meanwhile, other studies remind us that the dental practitioner should be alert to the risk of promoting serious mutilation of the jaw and, hence, to the face when performing a surgery in an exacerbated form of cherubism—recommending, especially in these patients, systemic calcitonin administration as an attractive and effective clinical therapy for larger lesion, relying on its effect of inhibiting the osteoclastic activity and, consequently, inhibiting of the bone resorption [14, 16, 21]. In this case report, the lesion reached worrisome proportions risking an imminent pathological fractures, besides thrive and speech impairment, and psychological disturbance, leading the team to opt for the immediate surgical intervention. 

In this case report, the patient was submitted to a conservative partial curettage of the lesion, and surgical defects were filled with autogenous cancellous bone and bone marrow grafts. The removal of the second molar involved in the lesion was not possible to execute during the surgery due to the risk of bone fracture, although its extraction will be executed on a further appointment. Several works point out the importance of an autogenous bone graft, which is an osteoinductive and osteoconductive material [18–21], promoting the acceleration of bone repair. Probably, This fact occurs due to the chemotactic, mitogenic, and osteogenic activity of the autogenous cancellous bone, and bone marrow grafts are associated with the presence of growth factors and autologous marrow-derived mesenchymal stem cells [19, 20, 22, 23]. We inferred that this could account for the presence of newly formed bone trabeculae in the graft areas, as well as the repairment of the remaining lesion areas by neoformed bone. Besides, the bone matrix is also rich in other growth factors such as bone morphogenetic proteins (BMPs), transforming growth factor-beta (TGF-beta), fibroblast growth factor (FGF), platelet-derived growth factor (PDGF), and epidermal growth factor (EGF) [19, 20, 22]. In addition, the salmon calcitonin was used concomitantly to inhibit multinucleated giant cells formation, osteoclast activity and proliferation, influencing in the regressive process of the lesion.

The biochemical examinations were within the normal range on our patient. However, it was demonstrated that alkaline phosphatase was increased, suggesting bone formation process and, consequently, remission of the lesion. 

**Figure 5:**
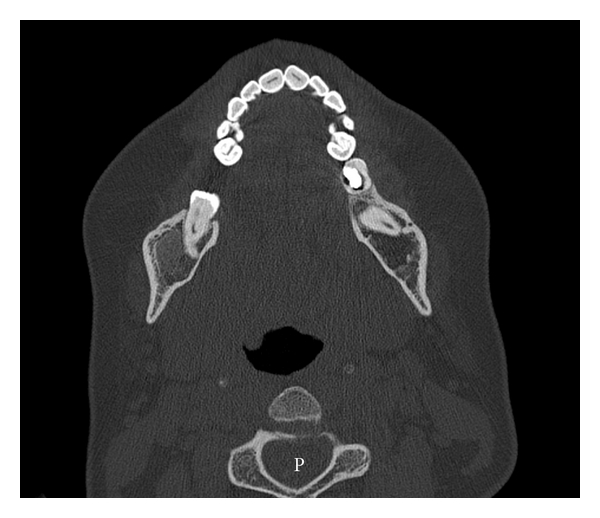
Axial CT scan showing significant regression of the intraosseous lesion with thick and preserved cortical bone at the angle and ramus of the mandible.

**Figure 6:**
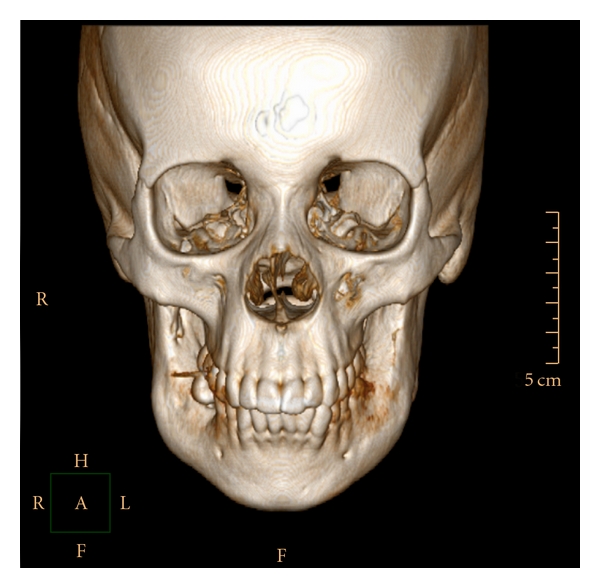
3D reconstruction of the CT scan images showing absence of depression and erosive areas at the mandibular body and retromolar region.

**Figure 7:**
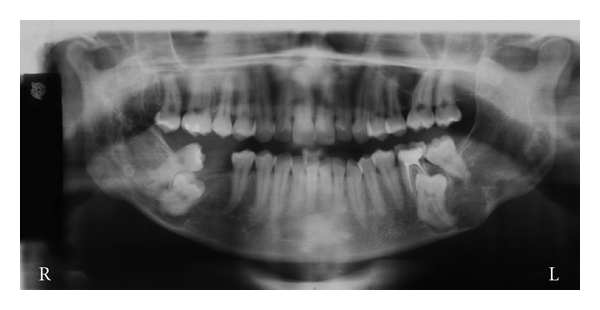
Panoramic radiograph revealing formation and replacement of the lesion by neoformed bone tissue, and the right mandibular ramus was larger than the left side.

In brief, this clinical case reports an aggressive form of cherubism whereas the surgical procedure was prescribed due to psychological and physiological damage installed. We assessed that the combination of the osteoinductive and osteoconductive material, as autogenous cancellous bone and bone marrow grafts, plus systemic calcitonin was effective in this aggressive cherubism's clinical management, building up strength to the bone repair process (Figures 5, 6, and 7). Despite the encouraging results obtained in this case report, we emphasize that future clinical studies are needed to address whether the combined therapy actually improved the healing process.

## References

[1] Hitomi G, Nishide N, Mitsui K (1996). Cherubism Diagnostic imaging and review of the literature in Japan. *Oral Surgery, Oral Medicine, Oral Pathology, Oral Radiology, and Endodontics*.

[2] Tiziani V, Reichenberger E, Buzzo CL (1999). The gene for cherubism maps to chromosome 4p16. *American Journal of Human Genetics*.

[3] Lannon DA, Earley MJ (2001). Cherubism and its charlatans. *British Journal of Plastic Surgery*.

[4] Ueki Y, Tiziani V, Santanna C (2001). Mutations in the gene encoding c-Abl-binding protein SH3BP2 cause cherubism. *Nature Genetics*.

[5] Ongole R, Pillai RS, Pai KM (2003). Cherubism in siblings: a case report. *Journal of the Canadian Dental Association*.

[6] Gomes MF, De Souza Setúbal Destro MF, De Freitas Banzi ÉC, Dos Santos SH, Claro FA, De Oliveira Nogueira T (2005). Aggressive behaviour of cherubism in a teenager: 4-Years of clinical follow-up associated with radiographic and histological features. *Dentomaxillofacial Radiology*.

[7] Yilmaz B, Ozan O, Karaagaclioglu L, Ersoy AE (2006). A prosthetic treatment approach for a cherubism patient: a clinical report. *Journal of Prosthetic Dentistry*.

[8] Raposo-Amaral CE, De Campos Guidi M, Warren SM (2007). Two-stage surgical treatment of severe cherubism. *Annals of Plastic Surgery*.

[9] Mangion J, Rahman N, Edkins S (1999). The gene for cherubism maps to chromosome 4p16.3. *American Journal of Human Genetics*.

[10] Faircloth WJ, Edwards RC, Farhood VW (1991). Cherubism involving a mother and daughter: case reports and review of the literature. *Journal of Oral and Maxillofacial Surgery*.

[11] Katz JO, Underhill TE (1994). Multilocular radiolucencies. *Dental Clinics of North America*.

[12] Whitaker SB, Singh BB (1995). Intraoral giant cell lesions: the peripheral and central forms of these entities. *Practical Periodontics and Aesthetic Dentistry*.

[13] Minic A, Stajcic Z (1996). Prognostic significance of cortical perforation in the recurrence of central giant cell granulomas of the jaws. *Journal of Cranio-Maxillo-Facial Surgery*.

[14] Southgate J, Sarma U, Townend JV, Barron J, Flanagan AM (1998). Study of the cell biology and biochemistry of cherubism. *Journal of Clinical Pathology*.

[15] Wada S, Udagawa N, Nagata N, Martin TJ, Findlay DM (1996). Calcitonin receptor down-regulation relates to calcitonin resistance in mature mouse osteoclasts. *Endocrinology*.

[16] de Lange J, Rosenberg AJWP, van den Akker HP, Koole R, Wirds JJ, Van Den Berg H (1999). Treatment of central giant cell granuloma of the jaw with calcitonin. *International Journal of Oral and Maxillofacial Surgery*.

[17] Claro FA, Lima JRS, Salgado MAC, Gomes MF (2005). Porous polyethylene for tissue engineering applications in diabetic rats treated with calcitonin: histomorphometric analysis. *International Journal of Oral and Maxillofacial Implants*.

[18] Gross JS (1997). Bone grafting materials for dental applications: a practical guide. *Compendium of Continuing Education in Dentistry*.

[19] Lieberman JR, Daluiski A, Einhorn TA (2002). The role of growth factors in the repair of bone biology and clinical applications. *Journal of Bone and Joint Surgery. Series A*.

[20] Szpalski M, Gunzburg R (2005). Recombinant human bone morphogenetic protein-2: a novel osteoinductive alternative to autogenous bone graft?. *Acta Orthopaedica Belgica*.

[21] Sengupta DK, Truumees E, Patel CK (2006). Outcome of local bone versus autogenous iliac crest bone graft in the instrumented posterolateral fusion of the lumbar spine. *Spine*.

[22] Block JE (2005). The role and effectiveness of bone marrow in osseous regeneration. *Medical Hypotheses*.

[23] Miura M, Miura Y, Sonoyama W, Yamaza T, Gronthos S, Shi S (2006). Bone marrow-derived mesenchymal stem cells for regenerative medicine in craniofacial region. *Oral Diseases*.

